# Disability insurance benefit application in Switzerland: an analysis of linked administrative and survey data

**DOI:** 10.1186/s12913-021-06992-2

**Published:** 2021-09-18

**Authors:** Szilvia Altwicker-Hámori

**Affiliations:** Institute of Health Sciences, School of Health Professions, ZHAW Zurich University of Applied Sciences, Katharina-Sulzer-Platz 9, 8401 Winterthur, Switzerland

**Keywords:** Disability insurance benefit application, Switzerland, Logistic regression

## Abstract

**Background:**

The guiding principle of disability insurance in Switzerland is ‘rehabilitation before pension’. Access to rehabilitation measures to restore, maintain or improve the earning capacity of individuals with disabilities is essential. Gainful employment enables them to be an active part of society, improves their quality of life, and may mitigate the adverse health effects of disability pension receipt. The aim of this study was therefore to identify factors for disability insurance benefit application in Switzerland.

**Methods:**

A novel dataset was created linking the 2010 Social Protection and Labour Market cross-section with administrative register data on disability insurance benefit application (2009–2018). Multiple logistic regression was employed to examine the associations between long-term health-related activity limitation, region of residence, demographic and socioeconomic characteristics and disability insurance benefit application in adults aged 18–55 (*N* = 18,448). Sensitivity analysis based on age was performed in individuals aged 18 to retirement age and aged 25 to 55.

**Results:**

The regression results showed higher odds of disability insurance benefit application for individuals suffering from long-term health-related activity limitations (OR 2.88; 95% CI 1.29–6.44; *p*-value 0.010); born outside of Switzerland (OR 1.75; 95% CI 1.32–2.32; *p*-value 0.000); living without a working partner (OR 1.54; 95% CI 1.17–2.02; *p*-value 0.002); living without a child aged 0–14 years (OR 1.70; 95% CI 1.29–2.26; *p*-value 0.000); aged 18–39 (OR 1.41; 95% CI 1.09–1.83; *p*-value 0.009); with a learnt occupation in ‘Manufacturing’ (OR 2.75; 95% CI 1.68–4.50; *p*-value 0.000), ‘Construction and mining’ (OR 2.03; 95% CI 1.13–3.66; *p*-value 0.018), ‘Trade and transport’ (OR 2.12; 95% CI 1.30–3.45; *p*-value 0.003), ‘Business and administration’ (OR 1.68; 95% CI 1.03–2.72; p-value 0.036), and ‘Health, teaching, culture and science’ (OR 1.55; 95% CI 1.05–2.29; *p*-value 0.026); and renters (OR 1.44; 95% CI 1.00–1.94; *p*-value 0.016). The results were robust to alternative samples defined by age – albeit with some differences in regional and learnt occupational patterns.

**Conclusions:**

The results suggested that disability insurance benefit application is more than a health-related phenomenon in Switzerland. However, the results provided a less consistent picture on the role of marginalization in application than in other European countries.

## Background

The guiding principle of disability insurance (DI) in Switzerland is ‘rehabilitation before pension’ [1, 2]. As such, individuals with disabilities or likely to become disabled are entitled to rehabilitation measures to restore, maintain or improve their earning capacity or their ability to perform day-to-day activities. Disability insurance benefits (DB) aimed at the rehabilitation of individuals with disabilities range from the provision of aids to integration and occupational measures. Only if the rehabilitation option has been exhausted is a person with disabilities entitled to a disability pension (DP) [2]. Application for DB is via the cantonal DI office of residence, either online or on paper. The 26 cantonal DI offices not only evaluate the degree of disability but also determine and monitor rehabilitation measures [1]. They are subject to expert, administrative, and financial supervision by the Federal Social Insurance Office (FSIO) [3].

The importance of ‘rehabilitation before pension’ is mirrored in the political agenda in Switzerland; in particular, in the three revisions to the Federal Act on DI between 2004 and 2012 [4–6]. The 4th revision (2004) introduced job placement services [6]; the 5th revision (2008) developed measures for early detection, early intervention, and integration in order to identify affected persons as early as possible and to support them in keeping their current jobs [7]; and the 6(a)^th^ revision (2012) was targeted at the labour market reintegration DP recipients [8]. During the reintegration period, DP-recipients continue to receive their benefits [8].

In light of the revisions and the guiding principle of DI, DB application may be considered a key stepping-stone to employment of individuals with disabilities; the importance of which cannot be overstated. Gainful employment enables individuals with disabilities to be an active part of society [9], improves their quality of life [10], may mitigate the adverse health effects of DP receipt [11–15], while increasing overall labour supply and economic output in the long-term [16]. Thus, identifying factors for DB application in Switzerland appears crucial and especially valuable when designing supportive measures for those eligible for rehabilitation but with barriers to DI.

Despite its significance, little is known on the factors for DB application. To the best of my knowledge, there are no Swiss studies. International evidence suggests that DB application behaviour is more than a medical phenomenon [17]; demographic and socioeconomic characteristics also play a role [18–21]. For example, high age, previous social assistance receipt, and low educational attainment were associated with higher odds of DP application between 1998 and 2004 in the Norwegian male and female population aged 18–66 in 1998 [18]. Being an upper-level non-manual employee and having more employment during the preceding four calendar years decreased the odds of applying for DP, whereas older age increased the odds of DP application in 2009 and 2014 in Finnish residents aged 18–64 in the respective years [20]. Furthermore, the Work Ability Index has been shown to be a predictor of DP application as well – albeit in a small and specific sample of individuals with chronic back pain living in Germany [22]. The short scale measuring the subjective prognosis of gainful employment (SPE-scale) has also been shown to be statistically significantly related to DP application in Germany in a cohort of blue-collar workers with low back pain or ‘functional syndromes of internal medicine’ [23].

Available international evidence cannot be applied to Switzerland given possible differences in local factors, such as labour market conditions, application processes as well as institutional settings; it merely informs the selection of potential factors. Therefore, the aim of the present study was to analyse the associations between long-term health-related activity limitation, region of residence, demographic and socioeconomic characteristics and DB application between 2009 and 2018 in adults living in Switzerland based on a novel dataset, linking the Social Protection and Labour Market (SESAM) data with administrative register data.

## Methods

### Data

Data for the statistical analysis was drawn from a linked dataset; linking the 2010 SESAM, provided by the Swiss Federal Statistical Office (FSO) [24, 25], with administrative register data, provided by the FSIO.

The SESAM is in itself a linked dataset. It merges microdata from the Swiss Labour Force Survey (SLFS) [26, 27], a telephone household survey carried out since 1991, and different social insurance registers; including ‘Old age, survivors’ and disability insurance’; ‘Disability pensions’; ‘Complementary benefits’; and ‘Unemployment insurance’ [25]. The respondents’ social insurance numbers serve as a key to link the data from registers to the SLFS data [25]. The SESAM has numerous advantages for the analysis. First, it combines detailed household survey data on educational background, financial status, and demographic characteristics with highly reliable longitudinal administrative data on registered unemployment and labour market measures for those seeking employment. Second, the SLFS adheres to international concepts and definitions, in particular to those employed in the European Union Labour Force Survey [28], thereby enabling international comparisons. Third, the SESAM has a relatively large sample size. It covers almost 1% of Switzerland’s permanent resident population aged 15 and over, corresponding to Swiss citizens whose main residence is in Switzerland and foreign citizens residing in Switzerland for at least 12 months [26].

However, the SESAM does not include information on DB application, the outcome variable. DB application was therefore retrieved from first-pillar social security registers for the time period of 2000 to 2018 and linked to the 2010 SESAM cross-section. The 2010 SESAM cross-section was chosen as (1) 2010 marks the first year when all independent variables were available in the SESAM and (2) it provides a sufficiently long DB-application history.

### Sample selection

The sample of interest was defined to include potential adult DB applicants between 2009 and 2018. Accordingly, individuals aged 18 to 55 in 2010 were included. Age 18–55 is a suitable age range: Age 18 represents the minimum age for ordinary DP entitlements in Switzerland [2]; age 55 ensures that even the oldest individuals in the sample are eligible for DB application for a sufficient time period. Within this sample, all DB applicants between 2000 and 2008 were excluded. The final sample included 18,448 individuals.

### Outcome variable

The outcome variable was a binary variable equal to one for DB application between 2009 and 2018 and zero otherwise.

### Independent variables

Our full model included information on long-term health-related activity limitation, sex, country of birth, household structure, age, learnt occupation, registered unemployment combined with labour market measure participation, homeownership, and region of residence. All independent variables were retrieved from the SLFS source of the SESAM, with the exception of registered unemployment and labour market measures, which were retrieved from the unemployment insurance register.

Long-term health-related activity limitation was captured by the Global Activity Limitation Indicator (GALI) [29]. The GALI belongs to the family of disability indicators [30]. It is a single-item self-reported survey instrument assessing health-related activity limitations and refers to general restrictions in activity without specifying the type of health problem or activity concerned (work, household chores, leisure personal care etc.) [30]. As such, the GALI shows the potential loss in the ability to assume expected social roles and to engage in regular activities that can affect the opportunities for social integration [31]. According to a recent study, the GALI as inclusive one question instrument fits all conceptual characteristics specified for a global measure on participation restriction and has a good and sufficient concurrent and predictive validity and reliability [32]. Moreover, it is worth noting that ‘subjective’ self-assessed health and disability measures have been found to be powerful predictors of DB application [33]. For the analysis, a dichotomous variable was generated to differentiate between those with and without long-term health-related activity limitation. The former category combined individuals who gave the answers ‘Severely limited’ or ‘Limited but not severely’ to the following question: ‘For at least the past six months, to what extent have you been limited because of a health problem in daily activities people usually do?’ [34].

Dichotomous variables for the respondent’s sex (‘Male’ versus ‘Female’), country of birth (‘Switzerland’ versus ‘Outside of Switzerland’), homeownership status (‘Homeowner’ versus ‘Renter’), the presence or absence of own children or step-children aged 14 years or younger and living in the same household were included. Similarly to a Swiss study on DP receipt [5], a dichotomous variable for the presence or absence of an employed partner in the household (cohabiting or married) was created combining (1) information on the relationship of household members to the survey respondent and (2) the respective household member’s employment status. The ‘Employed’ category included the following: employees, self-employed, apprentices, and family members working in family business. Age was dichotomised based on Erikson’s Stages of Psychosocial Development [35] to differentiate between young and middle adulthood (‘18–39’ versus ‘40–55’). The interest in the DB application odds of young adults relative to their middle-aged counterparts was mainly motivated by the notion that the labour market integration of young adults is especially important given the social and public health issues arising from labour market withdrawal or non-entry at young ages.

Learnt occupation in SESAM is categorised based on the Swiss Standard Classification of Occupations (SSCO) 2000 [36]. The SSCO 2000 classifies 20,000 occupations according to economic activity using five-digit codes. The five-digit occupations were aggregated at the highest level into ‘Divisions of professions’ (one-digit level) resulting in the following categories: (1) ‘Not applicable’; (2) ‘Agriculture, forestry, and livestock production’ [henceforth ‘Agriculture’]; (3) ‘Manufacturing’; (4) ‘Technical activities and ICT’, (5) ‘Construction and mining’; (6) ‘Trade and transport’; (7) ‘Hotels and catering, and other personal services [henceforth ‘Personal services’]; (8) ‘Management, administration, finance, insurance, and law’ [henceforth ‘Business and administration’]; (9) ‘Health, education, culture, and science’; and (10) ‘Not classifiable’. Note that the ‘Not applicable’ category included individuals who did not earn a specific occupational degree (they completed lower secondary education or general upper secondary education, for example).

The SESAM contains information on registered unemployment. More precisely, on the number of registrations with the Regional Employment Centre (RAV) as well as on participation in labour market measures (LMM) offered to those seeking employment in the five years preceding the survey. LMM aim to promote the rapid and long-term reintegration of insured persons into the labour market. They are designed to improve employability, strengthen professional qualifications in line with the needs of the labour market, reduce the risk of long-term unemployment, and allow insured persons to gain professional experience (for example, in the form of courses and internships) [37]. The above information was combined into three categories: (1) ‘No registrations with the RAV during the past five years’; (2) At least one registration with the RAV but no LMM during the past five years’; (3) ‘At least one registration with the RAV and at least one LMM the past five years’.

Finally, as set of indicator variables for region of residence were included, classified at the second Nomenclature of Territorial Units for Statistics (NUTS-2) level (‘Lake Geneva Region’, ‘Espace Mittelland’, ‘Northwestern Switzerland’, ‘Zurich’, ‘Eastern Switzerland’, ‘Central Switzerland’, and ‘Ticino’).

### Analysis

Characteristics of DB applicants and non-DB applicants were compared using Pearson’s chi-square tests. Multiple logistic regression models were applied to analyse the associations between long-term health-related activity limitation, region of residence, demographic and socioeconomic characteristics and DB application. Three models were estimated: Model 1 adjusted for long-term health-related activity limitation only; Model 2 adjusted for long-term health-related activity limitation and all demographic characteristics; and Model 3 (full model) adjusted for long-term health-related activity limitation, demographic as well as socioeconomic characteristics, and region of residence.

Sensitivity analysis was performed based on age. First, Model 3 was estimated in the full sample of individuals eligible for DB, that is, 18-to-63-year-old women and 18-to-64-year-old men (Model 4). Model 4 included three age group dummies (‘18–39’, ‘40–55’, and ‘56-retirement age’). Second, Model 3 was estimated in the subsample of 25-to-55-year-olds, corresponding roughly to individuals in their prime working lives (Model 5).

Further analyses, not reported in the present study, were carried out (available upon request). First, Model 3 was estimated using the subsample of 25-to-54-year-olds, corresponding to the most common definition of prime working age. The estimation results were in line with those aged 25–55. Second, Model 3 was estimated using DB applications between 2010 and 2018 only. This ensures that DB applications are observed after all socioeconomic information was collected in SESAM at the cost of excluding those who potentially applied due to their long-term health-related activity-limitations in 2009 (*N* = 18,414). The estimation results remained robust. Third, region of residence indicators classified at the NUTS-2 level were replaced by an indicator variable differentiating between rural and urban residential area (1 = ‘Rural’) in Model 3. No statistically significant association was found between residential area and DB application (OR 1.11; 95% CI 0.81–1.53; *p*-value 0.518) and the remaining estimates remained robust. In a further model, the residential area indicator was added to Model 3. No statistically significant association was found between residential area and DB application (OR 1.05; 95% CI 0.75–1.40; *p*-value 0.781) and the remaining estimates remained robust. Fourth, more detailed categories for long-term health-related activity limitation, differentiating between ‘severely limited’ and ‘limited but not severely’, were used in Model 3. The parameter estimated were statistically significant, indicating higher DB application odds for both groups relative to their counterparts without self-assessed activity limitaitons (OR 3.68; 95% CI 1.39–9.73; *p*-value 0.008 and OR 2.54; 95% CI 1.09–5.9; *p*-value 0.031, respectively); the remaining estimates remained robust.

Finally, further analyses were carried out to address the issue of the missing responses regarding long-term health-realated activity limitation. Before describing the results of these analyses, it is worth noting that using the complete case dataset with regard to long-term-health activity limitation means restricting the sample size to 3,142 observations. Whereas the inclusion of missing data as a dummy variable ensures a large enough sample for modelling, this reduction in sample size poses a problem given the distribution of the binary dependent variable. More specifically, the frequency counts in the cells of the two-way table are such that the estimation of the full model (Model 3), which is central to the study, is not advisable [38, 39]. Accordingly, only Models 1 and 2 using the complete case dataset with regard to long-term health-related activity limitations were estimated. The results regarding long-term health-related activity limitations remained robust. The results regarding the additional demographic variables in Model 2 indicated the same pattern as when using the full sample but were not statistically significant. The next set of analyses focused on the ‘missing’ subsample with regard to long-term health-related activity limitation (*N* = 15,293) in order to assess how the results in this subsample compare to those in the full sample. To this end, a model with the full set of independent variables except for long-term health-related activity limitation was estimated in two samples: (1) in the ‘missing’ subsample with regard to long-term health-related activity limitation and (2) in the full sample. The estimated parameters were in line with those of Model 3.

Categories for missing values (relevant only for the long-term health-related activity limitation, learnt occupation, and homeownership) were included in all analyses. Variance inflator factor was used to assess multicollinearity in all estimated models; there was no indication of multicollinearity. Results of the multiple logistic regression models are presented as odds ratios (OR), with 95% confidence intervals (95% CI) and *p*-values. A *p*-value of ≤5% was regarded as statistically significant in all analyses. All statistical analyses were carried out and reported in line with FSO regulations. Accordingly, individual weights, provided in the SESAM, were used in all analyses; statistics based on less than five observations are not reported; and statistics based on more than four but less than 50 observations are reported in brackets. In percentage calculations, the latter two FSO regulations apply to the numerator. All statistical analyses were conducted using Stata 15.

## Results

### Descriptive statistics

Table 1 describes the characteristics of respondents aged 18–55 by DB application. Around 10% of the full sample reported long-term health-related activity limitation; 7% reported none. Approximately half of the full sample was male (50%), was living with a working partner in the same household (51%), and was aged 18–39 (54%). The majority of the full sample was born in Switzerland (75%), did not have a child aged 0–14 years living in the same household (65%), was not registered as unemployed during the past five years (80%), and was not a homeowner (58%). For approximately 39% of the full sample learnt occupation was recorded as ‘Not applicable’. Around 18% had learnt an occupation within the field of ‘Health, education, culture, and science’, 9% in ‘Business and administration’, 8% in ‘Trade and transport’, 7% in ‘Manufacturing’, 7% in ‘Technical activities and ICT’, 4% in ‘Construction and mining’, 4% in ‘Personal services’, and 2% in ‘Agriculture’. For approximately 4% of the full sample the learnt occupation could not be classified. Approximately 19% of the full sample resided in the Lake Geneva Region, 22% in Espace Mittelland, 14% in Northwestern Switzerland, 18% in Zurich, 15% in Eastern Switzerland, 9% in Central Switzerland, and 4% in Ticino.

**Table 1:** Descriptive statistics for respondents aged 18–55 by DB application (weighted %)

	All (***N =*** 18,448)	Non-DB applicants (***n =*** 18,097)	DB applicants (***n*** = 351)	***p***-value
Long-term health-related activity limitation				
Yes	10.16	10.13	(11.38)	0.031
No	7.11	7.19	(2.89)	
Missing	82.74	82.68	85.73	
Sex				
Female	49.59	49.66	46.11	0.284
Male	50.41	50.34	53.89	
Country of birth				
Switzerland	74.79	75.01	63.85	< 0.001
Outside of Switzerland	25.21	24.99	36.15	
Working partner in household				
Yes	51.32	51.62	36.13	< 0.001
No	48.68	48.38	63.87	
Child aged 0–14 years in household				
Yes	34.64	34.90	21.52	< 0.001
No	65.36	65.10	78.48	
Age group				
18–39	54.28	54.04	66.59	< 0.001
40–55	45.72	45.96	33.41	
Learnt occupation				
Not applicable	38.69	38.87	29.55	0.049
Agriculture	1.85	1.87	X	
Manufacturing	7.04	6.93	(12.59)	
Technical activities and ICT	6.74	6.74	(6.90)	
Construction and mining	3.63	3.61	(4.79)	
Trade and transport	7.55	7.51	(9.55)	
Personal services	3.56	3.56	(3.82)	
Business and administration	8.79	8.78	(8.93)	
Health, education, culture, and science	17.71	17.71	17.75	
Not classifiable	4.43	4.42	(4.92)	
Registered unemployment and LMM during past 5 years				
No	80.21	80.29	76.16	0.009
Registered unemployment but no LMM	11.29	11.17	17.32	
Registered unemployment and LMM	8.50	8.54	(6.52)	
Homeowner				
Yes	41.40	41.68	27.12	< 0.001
No	58.08	57.81	71.36	
Missing	0.52	0.50	X	
Region of residence				
Lake Geneva Region	19.15	19.13	19.82	0.1064
Espace Mittelland	21.84	21.81	23.48	
Northwestern Switzerland	13.83	13.80	15.09	
Zurich	17.95	17.86	22.50	
Eastern Switzerland	14.53	14.63	(9.62)	
Central Switzerland	8.53	8.57	(681)	
Ticino	4.17	4.20	(2.69)	

*p*-values based on Pearson’s chi-square tests. Figures in brackets: Extrapolation based on less than 50 observations. The results should be interpreted with great caution. X: Extrapolation based on less than five observations. The results cannot be published for data protection reasons. DB: disability insurance benefit, LMM: labour market measures

There were statistically significant differences between non-DB applicants and DB applicants with regard to all the characteristics other than sex and region of residence. More non-DB applicants reported the absence of long-term health-related activity limitations (7%) than DB applicants (3%). There were more Swiss-born among non-DB applicants (75%) than among DB applicants (64%). In terms of household structure, a higher proportion of non-DB applicants was living with a working partner (52%) and with a child aged 0–14 years (35%) than DB applicants (36 and 22%, respectively). Non-DB applicants were on average older (46% versus 33% in the 40–55 cohort). More non-DB-applicants fell into to the category ‘Not applicable’ in terms of learnt occupation than DB-applicants (39 and 30%, respectively); and the opposite was true for ‘Manufacturing’ (7 and 13%, respectively). Registered unemployment within five years preceding the interview amounted to merely 20% in non-DB applicants and to 24% in DB applicants; homeownership to 42% and to just 27% in the respective groups.

The differences between non-DB applicants and DB applicants in the sample aged 18-retirement age and in the sample aged 25–55 showed the same pattern as in the 18–55-year-old group (results available upon request).

### Regression analysis

Individuals suffering from long-term health-related activity limitation were more likely (OR 2.79; 95% CI 1.25–6.22; *p*-value 0.012) to apply for DB than those without long-term health-related activity limitation (Table 2, Model 1). The results remained robust (OR 2.81; 95% CI 1.27–6.23; *p*-value 0.011 and OR 2.88; 95% CI 1.29–6.44; *p*-value 0.010, respectively) when adjusting for demographic and socioeconomic characteristics (Table 2, Models 2 and 3). Estimates of the full model (Table 2, Model 3) indicated that individuals born outside of Switzerland were more likely to apply for DB than those born in Switzerland (OR 1.75; 95% CI 1.32–2.32; *p*-value 0.000). Individuals without a working partner in the same household had higher odds of DB application (OR 1.54; 95% CI 1.17–2.02; *p*-value 0.002) than those living with a working partner. Individuals without a child aged 0–14 years living in the same household were also more likely (OR 1.70; 95% CI 1.29–2.26; *p*-value 0.000) to apply for DB than their counterparts living with a child aged 0–14 years. 18–39-year-olds showed higher odds of DB application than their 40–55-year old counterparts (OR 1.41; 95% CI 1.09–1.83; *p*-value 0.009). Individuals with a learnt occupation in ‘Manufacturing’ (OR 2.75; 95% CI 1.68–4.50; *p*-value 0.000), ‘Construction and mining’ (OR 2.03; 95% CI 1.13–3.66; *p*-value 0.018) ‘Trade and transport’ (OR 2.12; 95% CI 1.30–3.45; *p*-value 0.003), ‘Business and administration’ (OR 1.68; 95% CI 1.03–2.72; *p*-value 0.036), and ‘Health, teaching, culture and science’ (OR 1.55; 95% CI 1.05–2.29; *p*-value 0.026) had higher odds of DB application relative to those in the ‘Not applicable’ category. Renters were more likely (OR 1.44; 95% CI 1.0–1.94; *p*-value 0.016) to apply for DB than homeowners. Those residing in Eastern Switzerland had lower odds of DB application (OR 0.60; 95% 0.37–0.96; *p*-value 0.034) than their counterparts in Espace Mittelland. No statistically significant association was found between sex and DB application and registered unemployment and DB application.

**Table 2:** Logistic regression models with DB application as outcome, aged 18–55, *N =* 18,465 (weighted estimates)

	Model 1			Model 2			Model 3		
	OR	95% CI	***p***-value	OR	95% CI	***p***-value	OR	95% CI	***p***-value
Long-term health-related activity limitation									
No	1			1			1		
Yes	2.79	1.25–6.22	0.012	2.81	1.27–6.23	0.011	2.88	1.29–6.44	0.010
Sex									
Female				1			1		
Male				1.04	0.79–1.35	0.791	0.95	0.71–1.25	0.696
Country of birth									
Switzerland				1			1		
Outside of Switzerland				1.73	1.31–2.28	0.000	1.75	1.32–2.32	0.000
Working partner in household									
Yes				1			1		
No				1.48	1.12–1.96	0.006	1.54	1.17–2.02	0.002
Child aged 0–14 years in household									
Yes				1			1		
No				1.74	1.31–2.32	0.000	1.70	1.29–2.26	0.000
Age group									
40–55				1			1		
18–39				1.49	1.14–1.93	0.003	1.41	1.09–1.83	0.009
Learnt occupation									
Not applicable							1		
Agriculture							1.32	0.33–5.22	0.692
Manufacturing							2.75	1.68–4.50	0.000
Technical activities and ICT							1.50	0.90–2.52	0.120
Construction and mining							2.03	1.13–3.66	0.018
Trade and transport							2.12	1.30–3.45	0.003
Personal services							1.56	0.85–2.85	0.152
Business and administration							1.68	1.03–2.72	0.036
Health, teaching, culture, and science							1.55	1.05–2.29	0.026
Not classifiable							1.46	0.63–3.40	0.374
Registered unemployment and LMM in past 5 years									
No							1		
Registered unemployment but no LMM							1.29	0.88–1.89	0.192
Registered unemployment and LMM							0.68	0.43–1.07	0.096
Homeowner									
Yes							1		
No							1.44	1.07–1.94	0.016
Region									
Espace Mittelland							1		
Lake Geneva Region							0.88	0.58–1.33	0.547
Northwestern Switzerland							0.93	0.60–1.43	0.744
Zurich							1.02	0.68–1.54	0.907
Eastern Switzerland							0.60	0.37–0.96	0.034
Central Switzerland							0.74	0.45–1.23	0.252
Ticino							0.60	0.30–1.21	0.154

Dummy variables for missing vales were included in all models. DB: disability insurance benefit, LMM: labour market measures

The coefficient estimates remained robust to alternative samples (Table 3) with a few exceptions. In the sample aged 18 to retirement age (Model 4), those whose learnt occupation falls into the category ‘Personal services’ were more likely (OR 1.82; 95% CI 1.06–3.12; *p*-value 0.031) to apply for DB relative to those without occupational qualifications; and no statistically significant regional differences were found. Estimation results revealed higher DB application odds for individuals aged 40–55 relative to their counterparts aged 56 to retirement age (OR 1.60; 95% CI 1.11–2.31; p-value 0.011). In the sample aged 25–55 (Model 5), the estimate on ‘Business and administration’ was not statistically significant; and no statistically significant regional differences were found.

**Table 3:** Logistic regression models with DB application as outcome, aged 18-retirement age, *N =* 22,476; aged 25–55, *N =* 16,540 (weighted estimates)

	Model 4			Model 5		
	OR	95% CI	***p***-value	OR	95% CI	***p***-value
Long-term health-related activity limitation						
No	1			1		
Yes	2.43	1.25–4.73	0.009	3.59	1.57–8.21	0.002
Sex						
Female	1			1		
Male	0.94	0.72–1.23	0.652	0.96	0.70–1.33	0.815
Country of birth						
Switzerland	1			1		
Outside of Switzerland	1.73	1.32–2.27	0.000	1.69	1.24–2.29	0.001
Working partner in household						
Yes	1			1		
No	1.49	1.16–1.91	0.002	1.46	1.09–1.94	0.010
Child aged 0–14 years in household						
Yes	1			1		
No	1.68	1.27–2.22	0.000	1.61	1.20–2.16	0.002
Age group						
56-retirement age	1			1		
18–39	2.30	1.61–3.28	0.000	1.32	1.00–1.74	0.049
40–55	1.60	1.11–2.31	0.011			
Learnt occupation						
Not applicable	1			1		
Agriculture	1.24	0.32–4.88	0.754	1.10	0.22–5.60	0.907
Manufacturing	3.12	2.01–4.86	0.000	2.17	1.22–3.84	0.008
Technical activities and ICT	1.54	0.94–2.53	0.086	1.18	0.67–2.08	0.557
Construction and mining	2.16	1.25–3.74	0.006	2.26	1.20–4.26	0.012
Trade and transport	2.40	1.54–3.72	0.000	2.23	1.33–3.74	0.002
Personal services	1.82	1.06–3.12	0.031	1.54	0.80–2.97	0.198
Business and administration	1.75	1.11–2.75	0.016	1.29	0.78–2.15	0.325
Health, teaching, culture, and science	1.57	1.08–2.28	0.017	1.58	1.05–2.38	0.028
Not classifiable	1.48	0.64–3.44	0.357	0.56	0.10–2.95	0.491
Registered unemployment and LMM in past 5 years						
No	1			1		
Registered unemployment but no LMM	1.33	0.93–1.91	0.118	1.27	0.84–1.94	0.261
Registered unemployment and LMM	0.68	0.44–1.05	0.084	0.74	0.45–1.22	0.237
Homeowner						
Yes	1			1		
No	1.41	1.08–1.85	0.012	1.50	1.10–2.06	0.011
Region						
Espace Mittelland	1			1		
Lake Geneva Region	0.87	0.59–1.29	0.499	1.03	0.65–1.63	0.892
Northwestern Switzerland	0.93	0.62–1.40	0.730	0.98	0.61–1.56	0.925
Zurich	1.01	0.69–1.48	0.968	0.96	0.61–1.50	0.856
Eastern Switzerland	0.69	0.45–1.06	0.089	0.79	0.48–1.33	0.378
Central Switzerland	0.79	0.49–1.26	0.317	0.90	0.53–1.55	0.713
Ticino	0.62	0.33–1.19	0.154	0.51	0.23–1.10	0.087

Dummy variables for missing vales were included in all models. DB: disability insurance benefit, LMM: labour market measures

## Discussion

### Main findings

This study explored the associations between both medical and non-medical variables and DB application between 2009 and 2018 in adults aged 18–55 living in Switzerland using linked survey and administrative data. The estimation results suggested that those suffering from long-term health-related activity limitations, born outside of Switzerland, living without a working partner and without a child aged 0–14, of lower age, renting their homes had higher odds of DB application. Individuals residing in Eastern Switzerland had lower odds of DB application than their counterparts living in Espace Mittelland. Moreover, the results showed statistically significant associations between learnt occupation and DB application. The results were robust to alternative samples defined by age – albeit with some differences in regional and learnt occupational patterns.

Some of the present results are in line with international findings. However, caution is warranted in cross-country comparisons of DB application behaviour due not only to differences in modelling and samples under study but also due to differences in local factors, application processes, and institutional settings. For instance, adverse labour market conditions may bring in marginally qualifying or marginally interested applicants [40]; and the level of transaction costs encountered in the application process were found to affect application behaviour [41].

It is not surprising that in all estimated models self-reported long-term health-related activity limitation was associated with higher odds of DB application given that long-term inability to work or to carry out regular tasks as a result of a physical, psychological or mental impairment constitutes the very definition of disability by the DI and hence of DB entitlements in Switzerland [42]. Furthermore, the present results are in accordance with those of a US study indicating that ‘having at least one problem with instrumental activities of daily living’ is associated with DB application in men and women aged 18–64 [17]. The same study found a number of other health factors to be signicant factors of DB applications, such as ‘problem in lifting 10 lbs’ and ‘difficulty in walking a quarter of mile’ [17].

The results implying that those born outside of Switzerland were more likely to apply for DB than their counterparts born in Switzerland calls for more detailed analysis due to the heterogeneity of the non-Swiss-born group in this study. Among other things, cultural aspects, residence permits as well as the regulations governing social security between Switzerland and the respective country [1] may have an impact on DB application behaviour among those born outside Switzerland and should be taken into account in future analysis.

The finding that those living without a partner have higher odds of DB application is consistent with the US study according to which those married are less likely to apply for DB than those divorced or widowed [17]. A potential explanation for this finding involves financial stability due to the partner’s income which affects household labour force participation decisions [43, 44]. Those living without a working partner may have less financial stability and subsequently may be more likely to apply for DB than their counterparts living with a working partner. Moreover, it may reflect underlying household preferences and the economic theory of specialization [45–47]. The key assumption of the theory of specialisation is that individuals living in the same household pool their resources and maximise a joint utility function. Assuming that individuals are not equally productive in the labour market, the household’s utility can be maximised by specialisation in labour market work or domestic work (i.e. the partner with the higher earning capability engages in labour market work and the other specialises in domestic work). Empirically, the theory of specialisation has found support in labour market research in numerous European countries [45]. In the present study, DB application may be considered an opportunity to engage in labour market work. Applying the theory of specialisation would imply that the individual’s DB application is negatively associated with the partner’s labour market participation; hence underpinning the result that those without working partners have higher odds of DB application.

That individuals without a child aged 0–14 years had higher odds of applying for DB may reflect a negative effect of parenthood on labour supply and pursuing further education (which DB application may entail). In particular, numerous studies demonstrate that children have a negative effect on women’s labour supply [47]; and there is also evidence for reduced labour supply in fathers in Switzerland [48]. Although not directly comparable, the results of the US study imply a negative association between family size and DB application [17], while the Norwegian and Finnish studies do not control for such variables [18, 20]. This in turn suggests the importance of further analysing the association between parenthood and DB application and investigating its underlying mechanisms.

The negative association between age and DB application may seem intriguing in light of the international literature. The odds of applying increased with age in Finnish non-retired residents aged 18–64 [20], in Norwegian men and women aged 18–66 – albeit not in the oldest age group – [18], and in those aged 18–64 in the US [17]. Furthermore, the results may seem surprising given that higher age, an indicator of health status, has been widely documented to be a risk factor for DP in the working-age population [49–52]. One possible explanation for the findings in the present study involves the ‘lifetime perspective’. Young adults may gain the most from the ‘rehabilitation before pension’ principle behind DI, which in turn may explain their higher odds of DB application. Qualitative research may be valuable in investigating this potential explanation.

The results indicate that especially those in physically demanding learnt occupations, such as construction [53], had higher odds of DB application. This is in line with the Finnish study arguing that physically straining occupations generate a high risk of occupational disability; in many such occupations an employee with a physical condition cannot execute work tasks at all and applies for a rehabilitation programme or pension [20]. It is also in line with the US study showing that significantly more DB applicants come from occupations classified as hazardous, blue-collar or having a strength requirement [17]. Those in typically sedentary, white-collar occupations, such as business and administration, also showed higher DB application odds; a result not surprising in light of the evidence suggesting sedentary time to be associated with negative health issues [54]. Note that a Swiss study on the DP receipt in young adults found high odds of DP receipt in the above mentioned learnt occupational groups [5].

The higher application odds for renters are not surprising. In Switzerland, homeownership is low in international comparison due not only to high prices but also to a restrictive mortgage system and high down-payment requirements [55]. Therefore, the homeownership variable is likely to reflect individual and household wealth; and as such the findings are consistent with international studies. For example, health-impaired adults with high financial assets were found to be less likely to apply for DB in the US [17].

In a well-functioning social security system, we would not expect a relationship between DB application and registered unemployment per se. Therefore, it is not surprising that the coefficient estimates on the variables capturing past registered unemployment with and without LMM participation were not significant at conventional levels. Nevertheless, they are worth discussing in the context of mixed available evidence regarding employment history. The US study spent considerable effort testing the significance of a number of unemployment/lay off variables prior to DB application, but these individual-level employment-status variables were not significant in the DB application regressions [17]. The Norwegian study on the other hand found high odds of DP application among men with marginal or no work affiliation compared to those with stable affiliation; this tendency was less pronounced among women [18]. Similarly, the Finnish study found evidence that being employed less than 50% of the four preceding calendar years increased the odds of DP application; the effect decreased slightly between 2009 and 2014 [19]. According to the authors, one explanation for this finding may lie in a higher incentive for seeking financial security via DP in those with worse work histories. This in turn aligns with their hypothesis regarding marginalisation – being in one undesirable state of partial participation in one or more areas of society –, according to which for a marginalised person, DP may seem as a shelter from social insecurity, thus giving rise to potentially ‘unjustified’ applications [18]. The findings in the present study do not align with the concept of marginalisation; those with unemployment registrations, independent of LMM participation, were not more likely to apply for DB over the period under analysis. Nevertheless, LMM participation may be deemed important to control for in future research given that successful LMM may lead to better employment possibilities, rendering DP application unnecessary in the context of marginalisation.

### Methodological considerations

A strength of this study is the use of a novel dataset linking administrative and survey data. Such linkage is a promising and innovative strategy as it combines highly reliable administrative records with detailed survey information that is essential for the statistical analysis [56]. Furthermore, unlike most studies which are claimed to be restricted on gender/age dimensions due to limitations in sample design [17], the present study is inclusive along both dimensions and contains robustness checks along the age dimension. Most importantly, to the best of my knowledge, by merging the appropriate datasets, it was possible to explore the factors for DB application for the first time in Switzerland, thereby not only generating novel results but also demonstrating the potential of this innovative dataset for the research question at hand.

Nevertheless, the dataset does have its limitations. First, the activitly limitation measure had a lot of missing values. Second, the advantage of a long potential application period comes at the cost of not capturing the potential occurrence of health-related activity limitations after 2010. Third, similarly to other studies [18, 20], the dataset did not allow to control for medical diagnosis. Integrating broad medical diagnosis groups in the analysis of DB application would be useful in light of the evidence indicating that indivuduals with ‘congenital medical conditions’ and ‘acute medical conditions’ are more likely to apply for DB than their counterparts without these conditions in the US [17].

Moreover, a more comprehensive measure of unemployment would be beneficial in future analysis given that a substantial proportion of the unemployed in Switzerland were not registered with a RAV for the study period [57]. To this end, panel data with long and large panels containing information on unemployment as defined by the International Labour Organization (ILO) would be an asset as the ILO figures also include the unemployed who are not registered with the RAV [58]. In addition, studying under-employment in this context would be valuable.

Furthermore, the present study is based on individuals participating in the SLFS in 2010. An analysis of an additional, more recent SLFS cross-section would hence be desirable to check the robustness of the present results, once the relevant DB application data is available. In addition, because of the data and method at hand, caution is warranted with causal interpretation. Many of these limitations apply to the general context of DB research in Switzerland [5]; hence future research should centre around overcoming these limitations by using even better data. Future research is also warranted focusing on the group of DB applicants in order to generate insights on their employment prospects in view of the Swiss DI’s guiding principle of ‘rehabilitation before pension’. Finally, in terms of methodology, a time-to-event analysis may represent a valuable extension to the present analysis.

## Conclusions

The results suggested that DB application is more than a health-related phenomenon in Switzerland. Demographic and socioeconomic characteristics were also associated with DB application. As such, this study confirmed international research findings. However, the results provided a less consistent picture on the role of marginalization in DB application than in other European countries; suggesting that DI was not a ‘reservoir’ for marginalised individuals within the Swiss social insurance system for the period under analysis. From a policy perspective, the findings regarding long-term health-related activity limitation and occupation reaffirmed the importance of well-targeted rehabilitation measures in order to achieve an optimal match between individual vulnerability and occupational characteristics [5, 59].

## Data Availability

The data that support the findings of this study are available from the Swiss Federal Statistical Office and the Swiss Federal Social Insurance Office, but restrictions apply to the availability of these data, which were used under license for the current study, and so are not publicly available.

## Abbreviations

DI: Disability insurance
DB: Disability insurance benefit
DP: Disability pension
SLFS: Swiss Labour Force Survey
FSIO: Swiss Federal Social Insurance Office
SESAM: Social Protection and Labour Market
FSO: Swiss Federal Statistical Office
GALI: Global Activity Limitation Indicator
SSCO: Swiss Standard Classification of Occupations
RAV: Regional employment centre
NUTS-2: Nomenclature of Territorial Units for Statistics
OR: Odds ratio
95% CI: 95% confidence interval
ILO: International Labour Organisation
